# Identifying similarity- and rule-based processes in quantitative judgments: A multi-method approach combining cognitive modeling and eye tracking

**DOI:** 10.3758/s13423-024-02624-y

**Published:** 2025-02-25

**Authors:** Florian I. Seitz, Rebecca Albrecht, Bettina von Helversen, Jörg Rieskamp, Agnes Rosner

**Affiliations:** 1 Center for Economic Psychology, Department of Psychology, University of Basel, Missionsstrasse 62a, 4055 Basel, Switzerland; 2 Center for Cognitive Science, Department of Psychology, University of Freiburg, Freiburg, Germany; 3 Department of Psychology, Faculty of Human and Health Sciences, University of Bremen, Bremen, Germany; 4 Cognitive Psychology Unit, Institute of Psychology, Leibniz University Hannover, Hannover, Germany; 5 Cognitive Psychology Unit, Department of Psychology, University of Zurich, Zurich, Switzerland

**Keywords:** Judgment, Decision-making, Eye tracking, Computational modeling, Looking-at-nothing, Exemplar

## Abstract

Quantitative judgments have been suggested to result from a mixture of similarity- and rule-based processing. People can judge an object’s criterion value based on the object’s similarity to previously experienced exemplars and based on a rule that integrates the object’s cues like a linear regression. In order to better understand these processes, the present work combines cognitive modeling and eye tracking and tests whether people who rely more on the similarity to exemplars also look more at the exemplar locations on the screen. In two eye-tracking studies, participants learned to assign each of four exemplars to a different screen corner and criterion value and then judged the criterion value of briefly presented test stimuli. Eye tracking measured participants’ gazes to the now empty exemplar locations (a phenomenon called *looking-at-nothing*); cognitive modeling of the test phase judgments quantified participants’ reliance on a similarity- over a rule-based process. Participants showed more similarity use and more looking-at-nothing in the study in which the cues were linked to the criterion by a multiplicative function than in the study with an additive cue-criterion link. Focusing on the study with a multiplicative environment, participants relying more on the similarity to exemplars also showed more looking-at-nothing (${\tau }$ = 0.25, *p* = .01). Within trials, looking-at-nothing was usually directed at the one exemplar that was most similar to the test stimulus. These results show that a multi-method approach combining process tracing and cognitive modeling can provide mutually supportive insights into the processes underlying higher-order cognition.

The human mind relies on similarities and rules to predict unknown values – be it to infer a discrete criterion in categorization (e.g., Is this job applicant suitable?; Rouder & Ratcliff, 2006; Smith & Sloman 1994) or a numeric criterion in quantitative judgment (e.g., How suitable is this job applicant?; Juslin, Olsson, & Olsson, 2003; von Helversen & Rieskamp, 2009). People may assign similar objects to similar criterion values or apply a rule to predict an object’s criterion value from its cues. For example, the suitability of job applicants can be judged based on their similarity to hired employees or by integrating their various skills. People often rely both on similarity- and rule-based processes (Albrecht, Hoffmann, Pleskac, Rieskamp, & von Helversen, 2020; Erickson & Kruschke, 1998; Bröder, Gräf, & Kieslich, 2017), and even in tasks that favor one process, the other process can affect people’s responses (Hahn, Prat-Sala, Pothos, & Brumby, 2010; Rosner & von Helversen, 2019; von Helversen, Herzog, & Rieskamp, 2014). However, identifying to what extent people rely on similarity or a rule can prove difficult (see Hahn & Chater, 1998): The two processes make similar response predictions in many tasks (e.g., Nosofsky, Clark, & Shin, 1989; Rouder, & Ratcliff, 2006; Juslin et al., 2003) and are sometimes thought to constitute the extremes on a continuum that allows for fine-grained mixtures (e.g., Pothos, 2005; Bröder et al., 2017). The present work investigates similarity- and rule-based processes in quantitative judgments from a novel angle by combining two methodological approaches: cognitive modeling to identify the two processes at the response level and eye tracking for a better understanding of the process level.

## Identifying similarity- and rule-based processes

We establish the link between cognitive modeling and eye tracking via the role memory retrieval plays when people infer an object’s criterion value. Specifically, the similarity-based process relies on the object’s similarity to previously experienced objects called *exemplars* (Nosofsky, 1986; Juslin et al., 2003) which are retrieved from episodic memory (Hoffmann et al., 2014)^1^. The more the mind relies on similarity over rules, the more it relies on exemplar retrieval from memory. Memory retrieval, in turn, has been linked to eye movements that reinstate the encoding context: Fixating the spatial location in which some information was previously encoded can help recalling this information (Wynn, Shen, & Ryan, 2019). Accordingly, when people retrieve an exemplar from memory, they tend to fixate on the spatial location that the exemplar occupied during encoding (e.g., Scholz, von Helversen, & Rieskamp, 2015). This suggests a close link between cognitive modeling, which quantifies the reliance on similarity over rules, and eye tracking, which measures people’s gaze proportions to the exemplars. The present work puts this alleged link to the test.

### Cognitive modeling

When similarity- and rule-based processes make distinct predictions, cognitive modeling provides a way to identify them by measuring their relative contributions to people’s responses. For instance, the RulEx-J model of Bröder et al. (2017) predicts a person’s judgment $\hat{c}_i$ for object *i* as a weighted average of the predictions $\hat{c}_i^{\text {Similarity}}$ and $\hat{c}_i^{\text {Rule}}$ of the similarity- and rule-based processes (Izydorczyk & Bröder, 2022, 2023). Formally,

1 $$\begin{aligned} \hat{c}_i = \alpha \cdot \hat{c}_i^{\text {Similarity}} + (1 - \alpha ) \cdot \hat{c}_i^{\text {Rule}}, \end{aligned}$$

where $\alpha$ is a free parameter (with $0 \le \alpha \le 1$) representing a person’s relative reliance on the similarity- over the rule-based process. Specifically, $\alpha$ can reflect the weight with which one combines the two processes within trials or the probability with which one relies on one of the two processes in a trial (Bröder et al., 2017).

**Fig. 1:**
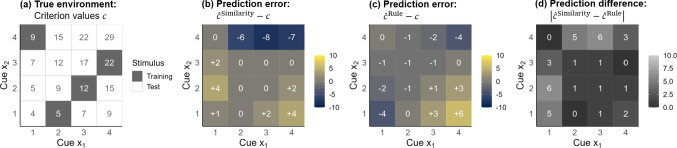
Multiplicative environment used in the main study. Participants estimated a numeric criterion *c* of stimuli with two multivalued cues $x_1$ and $x_2$. (**a**) The true criterion values resulting from $c = \lfloor {(\frac{5}{3} \cdot x_1 \cdot x_2) + 2\rceil }$, where $\lfloor$ and $\rceil$ round the criterion values to the closest integer. Further shown are the prediction errors from (**b**) a similarity-based process and (**c**) a rule-based process, and (**d**) the absolute prediction differences between the two processes. The predictions stem from processes that optimally learned the criterion values of the four shaded cue combinations in (**a**) that served as exemplars in the experiment (i.e., the rule- and similarity-based processes that minimize the prediction error on the four exemplars were used to predict judgments for all stimuli). In the experiment, participants learned the exemplars’ criterion values in a training phase with feedback and then judged the criterion values of all stimuli in a test phase without feedback

Typically, $\hat{c}_i^{\text {Similarity}}$ and $\hat{c}_i^{\text {Rule}}$ are computed based on Juslin et al. (2003, for the equations, see Appendix A): The similarity-based process predicts an object’s criterion value to be the mean of the exemplars’ criterion values, each weighted by the exemplar’s normalized similarity to the object. Rooted in categorization (Nosofsky, 1986), this similarity-to-exemplars approach successfully describes judgments (e.g., Albrecht, Jenny, Nilsson, & Rieskamp, 2021; Juslin et al., 2003), notably when the cues predict the criterion in a non-linear (e.g., multiplicative) way (e.g., Karlsson, Juslin, & Olsson, 2007; Juslin, Karlsson, & Olsson, 2008; Hoffmann, von Helversen, & Rieskamp, 2013; Mata, von Helversen, Karlsson, & Cüpper, 2012). The rule-based process, in turn, computes a weighted sum of the cue values like a linear model and can predict people’s judgments especially in linear, additive environments. The reason for assuming additive rules are capacity limitations that often hinder the cognitive system from learning more complex rules (Juslin et al., 2008; Brehmer, 1994, 1969). Compared to the similarity process, rules readily extrapolate beyond the range of the exemplars’ criterion values; therefore, the two processes can notably be distinguished using stimuli with extreme cue values. Figure 1 shows the two processes’ prediction errors for a multiplicative environment. For the stimuli with extreme cue values, the similarity-based predictions are too moderate (Fig. 1b), while the rule-based predictions can even be too extreme (Fig. 1c), leading to large prediction differences (Fig. 1d).

### Eye tracking

Another way to distinguish similarity- from rule-based processes are process tracing methods such as eye tracking. Specifically, when people retrieve some information from memory they tend to look at spatial locations associated with the information during encoding – although the information is no longer visible (called *looking-at-nothing*, Ferreira, Apel, & Henderson, 2008; Richardson & Spivey, 2000; Scholz et al., 2015; Spivey & Geng, 2001; Richardson, Altmann, Spivey, & Hoover, 2009; Renkewitz & Jahn, 2012). For instance, when asked to recall a geometrical shape’s orientation or color, participants fixated the blank area on the computer screen in which the shape was originally presented in 24% of all trials (Spivey & Geng, 2001). Cognitive explanations for looking-at-nothing are that eye movements to a spatial location facilitate the memory retrieval of information encoded at that location (Wynn et al., 2019) or reflect attention shifts to information in memory (Scholz, Klichowicz, & Krems, 2018).

Applied to criterion inference tasks, looking-at-nothing is found during exemplar retrieval and may thus be related to similarity-based processing (Rosner & von Helversen, 2019; Scholz et al., 2015; Rosner, Schaffner, & von Helversen, 2022). For instance, compared to a condition that instructed the use of a rule, a corresponding similarity condition led people to fixate longer on blank screen areas previously associated with one exemplar each (Scholz et al., 2015). This difference was particularly pronounced when an exemplar matched the to-be-judged object: The participants in the similarity condition fixated the corresponding spatial position for about 2 s and about 1.5 s longer than the participants in the rule condition. Furthermore, looking-at-nothing can predict people’s responses both for quantitative judgments (Rosner & von Helversen, 2019) and categorizations (Rosner et al., 2022), suggesting a tight link to the cognitive processes underlying human inferences.

## Overview and research aim

The present work extends the previous findings by adding cognitive modeling to test if looking-at-nothing can distinguish similarity- from rule-based processing. Specifically, we test if parameter $\alpha$ reflecting a participant’s reliance on a similarity process in cognitive modeling correlates with the gaze proportions to the blank exemplar locations. Additionally, we investigate the properties of such looking-at-nothing, analyzing at what time in a trial it occurs, the number of exemplars looked at per trial, and their similarity to the object being judged. To this end, we ran two eye-tracking studies using a multiple-cue judgment task. Participants learned the criterion values and screen locations of four exemplars in a training phase with feedback and then judged the criterion value of briefly presented test stimuli without feedback. One study defined the criterion by a linear, additive function; the other study by the non-linear, multiplicative function of Fig. 1.

Much more looking-at-nothing was observed in the multiplicative environment than in the additive environment. This is consistent with our predictions, as multiplicative environments tend to be associated with similarity use and additive environments with rule use (Karlsson et al., 2007; Juslin et al., 2008). Yet, the low looking-at-nothing rates in the additive environment limit its informative value. Therefore, we report the findings from the additive environment in Appendix B and the findings from the multiplicative environment with substantial looking-at-nothing below in the main text.

**Fig. 2:**
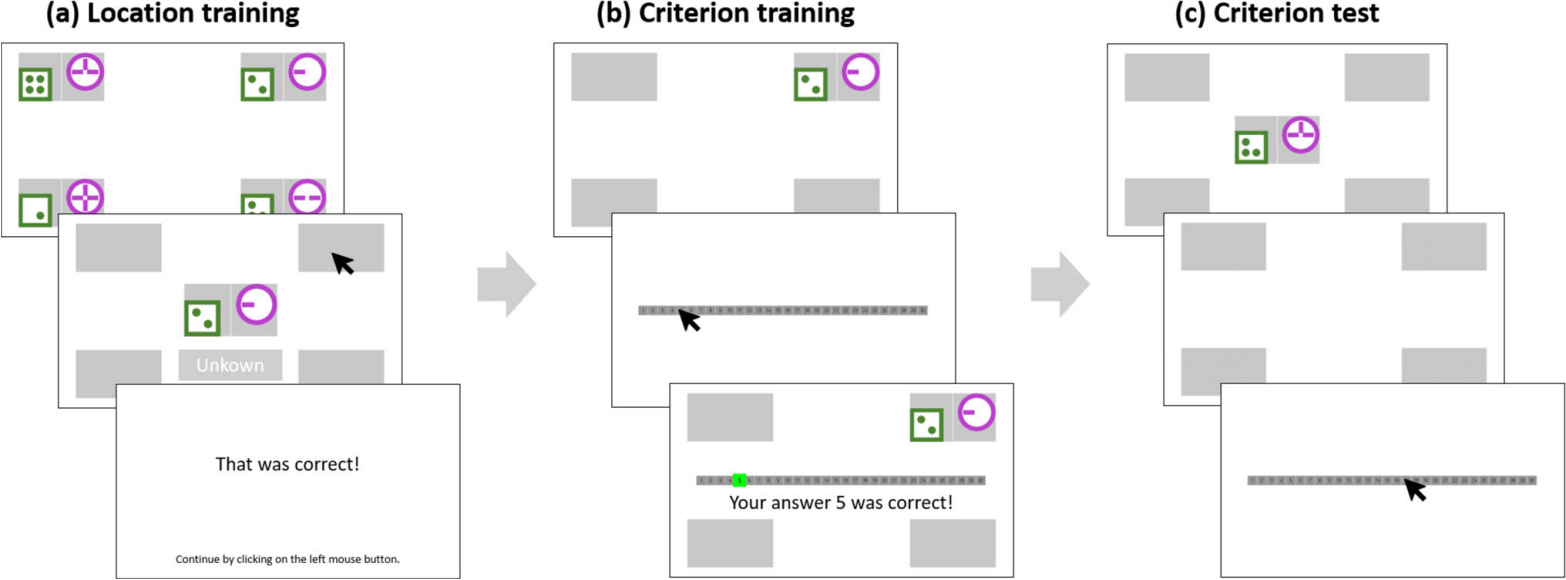
Experimental procedure. In location training (**a**), participants first saw all four exemplars at their respective location; then, they saw one stimulus per trial, clicked on the associated location or the unknown button, and got feedback. In criterion training (**b**), participants saw an exemplar, clicked the left mouse button to reach a scale, entered their judgment, and got feedback. In criterion test (**c**), participants briefly saw a stimulus in the screen center. The exemplar locations were shown as *gray rectangles* until participants clicked the left mouse button to enter their judgment. There was no feedback in the criterion test. Eye tracking measured participants’ gazes to the blank exemplar locations after the test stimulus removal (looking-at-nothing), and the criterion test responses were used for cognitive modeling

## Study with a multiplicative environment

### Method

#### Participants

In total, 56 students from the University of Zurich participated in a laboratory eye-tracking experiment in exchange for CHF 15.00 ($\approx$ USD 17.00) or course credit. Seven participants did not complete the experiment due to eye-tracking problems, and one participant was excluded for assigning larger criterion values to objects with smaller cue values – a response pattern opposite to our true environment (Fig. 1) and incompatible with our modeling framework (App. A). This led to a final sample of *N* = 48 participants (31 women; age: *M* $\pm$ *SD* = 26 $\pm$ 8 years). Assuming a type I error of .05 and a power of .95, *N* = 48 allows to detect a correlation of $\rho$ = .49 between looking-at-nothing and parameter $\alpha$ from Eq. 1, as a sensitivity power analysis for a two-sided bivariate correlation revealed^2^. The experiment took on average about 1 hour and was approved by the ethics board of the Faculty of Arts and Social Sciences of the University of Zurich.

#### Apparatus

The experiment was programmed in Expyriment (Krause & Lindemann, 2014) using Python 2.7 (Van Rossum & Drake Jr, 1995). Participants were seated in front of a 22-in. computer screen (1920 $\times$ 1080 pixels) at a distance of 700 mm and instructed to position their heads in a chin rest. The eye-tracker system SMI iView RED sampled data from the right eye at 500 Hz and recorded with iView X 2.8 following a five-point calibration. Fixation detection was done with IDF Event detector 9 (SMI, Teltow) using a peak velocity threshold of 30$^\circ$/s and a minimum fixation duration of 80 ms.

#### Design and materials

The stimuli were geometrical figures consisting of two cues ($x_1$ and $x_2$) with four possible values each (from 1 to 4) and a criterion value *c* defined by the multiplicative function $c = \lfloor {(\frac{5}{3} \cdot x_1 \cdot x_2) + 2\rceil }$, where $\lfloor$ and $\rceil$ round the criterion value to the closest integer. Figure 1 (a) shows the possible 16 cue combinations and their true criterion values. Four of the 16 stimuli were selected as exemplars (the shaded cue combinations in Fig. 1a). These stimuli were selected so that both cues take on every possible value exactly once and that some of the remaining stimuli extrapolate beyond the range of the exemplars. Figure 1 (b) and (c) show the prediction errors of the similarity- and the rule-based processes, when trained on the four exemplars. Figure 1 (d) shows that the two processes make different predictions in particular for stimuli 11 (meaning $x_1 = 1$ and $x_2 = 1$), 12, 34, and 44, but not for stimuli close to multiple exemplars. One cue was a rectangle containing 1–4 dots, the other cue was a circle containing 1–4 lines. The two cues were presented side by side on the screen; one cue was presented in green, and the other in purple (see Fig. 2 for examples). The cue-color association and the cue-side association within stimuli were randomized across participants.

Each of the four exemplars was assigned to one screen corner, with a randomized stimulus-corner association across participants. The distance from the screen center to the center of each exemplar was 9.59$^\circ$ of visual angle (477 pixels; 415 pixels on the *x*-axis and 235 pixels on the *y*-axis). Each exemplar had a size of 6.39$^\circ$ $\times$ 3.69$^\circ$ of visual angle (320 $\times$ 180 pixels). For the gaze analyses, we drew four rectangular areas of interest (AOIs) around the exemplar locations and one around the center of the screen where the test stimuli were presented. Each AOI had a size of 7.67$^\circ$ $\times$ 4.43$^\circ$ of visual angle (384 $\times$ 216 pixels). The size of the AOI exceeded the outer borders of each stimulus rectangle by a factor of 0.1, equaling 0.64$^\circ$ of visual angle (32 pixels) on the *x*-axis and 0.37$^\circ$ (18 pixels) on the *y*-axis.

#### Procedure

Participants’ task was to judge the criterion values of stimuli on a scale from 1 to 30. Participants completed a training phase in which they learned the location and criterion value of four exemplars and a test phase in which they judged the criterion value of all 16 possible stimuli. In the test phase, the stimuli were only briefly presented, and we measured looking-at-nothing after stimulus removal by recording participants’ eye movements to the four corners of the screen associated with the exemplars in the training phase. The resulting experimental procedure is illustrated in Fig. 2 and detailed below.

In the location training phase (see Fig. 2a), participants learned to assign the four exemplars to the four corners of the screen within a maximum of 15 blocks. Each block contained eight stimuli – the four exemplars and four distractors with reversed cue values (e.g., distractor 12 for exemplar 21), with random order within blocks. At the beginning of each block, participants could study the four exemplars at their associated screen locations. Then the locations turned to gray rectangles, and participants indicated for one stimulus after another the correct location by clicking on the associated rectangle. If a stimulus was a distractor, participants should click on an “unknown” button in the lower part of the screen. Location training ended after participants answered all stimuli correctly in three consecutive blocks or after 15 blocks.

In the criterion training phase (see Fig. 2b), participants learned to assign the four exemplars to their criterion values. The four stimuli were presented in random order within each block for a maximum of ten blocks or until participants answered all four stimuli correctly in three blocks. In each trial, participants studied one exemplar in its associated location without time limit (the locations of the other exemplars remained gray). Then, they pressed the left mouse button and reached a scale ranging from 1 to 30 on which they entered their numeric judgment within 2 s. A correct response was highlighted in green; an incorrect response was highlighted in red together with the correct value highlighted in green. Additionally, verbal feedback was presented below.

In the critical criterion test phase (see Fig. 2c), participants judged the criterion value of all 16 possible stimuli in eight blocks (128 trials), with random order within blocks. In each trial, participants saw a briefly presented test stimulus in the middle of the screen and the four gray rectangles representing the locations of the exemplars, which remained visible after the test stimulus was removed from the screen. After the test stimulus removal, eye tracking measured participants’ gaze durations to the blank exemplar locations until they pressed the left mouse button (without time limit). Participants then entered their judgment on the response scale within 2 s and got no feedback.

The test stimulus presentation time was individually calibrated at the beginning of the experiment in a psychophysics test, following a step-wise procedure recommended by García-Pérez (1998): In each trial of the psychophysics test, the participants saw a stimulus in the middle of the screen for a certain duration. Afterwards, four comparison stimuli were each presented in the corner of the screen, and the participants indicated which one corresponded to the original stimulus. The three non-matching alternatives were selected randomly, subject to the constraint that two of them matched the original stimulus in one cue each. The presentation duration started with 2000 ms and was decreased by 336.60 ms after four correct answers in a row and increased by 400 ms after a mistake (a 4-down-1-up staircase method). All 16 stimuli were shown in random order in three blocks (48 trials), and the mean across blocks yielded the stimulus presentation time in the test phase (*M* = 412 ms and *SD* = 220 ms across participants).

At the end of the experiment, a location test was conducted to check if the participants were still able to recall the exemplar locations. The location test had the same procedure as location training except that it comprised only one block (eight trials), that no feedback was given, and that in the beginning, no picture with all exemplars was shown.

#### Cognitive modeling

The RulEx-J model parameters were estimated by maximum likelihood from individual participants’ test phase data using a 16-fold cross-validation and were averaged using the mean by participant across folds^3^. The RulEx-J model contained five free parameters ($\alpha$ from Eq. 1, two cue weights $\beta _1$ and $\beta _2$, an intercept $\beta _0$, and a standard deviation $\sigma$, see Appendix A for details and Appendix C for the resulting estimates). Appendix A also shows that $\alpha$ was well recovered in our task, allowing to correlate participants’ $\alpha$ estimates with their looking-at-nothing. Appendix C further presents an out-of-sample model comparison between the RulEx-J, a pure similarity model (implying $\alpha$ = 1), and a pure rule model (implying $\alpha$ = 0).

**Table 1:** Variable distributions, normality coefficients *W*, and Kendall’s $\tau$ correlation coefficients

				Normality		Correlations: $\uptau$ and *p*			
Variable	*M*	*Mdn*	*SD*	*W*	*p*	1	2	3	4
1. Exemplar accuracy	.78	.88	.22	.88	< .001		< .001	< .001	< .001
2. Looking-at-nothing: duration	.19	.11	.20	.85	< .001	.34		< .001	.01
3. Looking-at-nothing: strength	.20	.13	.21	.86	< .001	.39	.84		.001
4. Cognitive modeling: $\alpha$ value	.40	.34	.32	.91	.001	.40	.25	.33	

*Note.* *W* denotes the statistic from a Shapiro–Wilk test against normality. The correlation table shows $\uptau$-coefficients below the diagonal and *p* values above the diagonal. Looking-at-nothing: duration equals the gaze duration to all exemplar locations divided by the gaze duration to the exemplar and test stimulus locations; strength equals the gaze duration to the location whose exemplar is most similar to the test stimulus divided by the gaze duration to all exemplar locations

### Results

The correlation analyses reported below use Kendall’s $\tau$ due to normality violations in participants’ looking-at-nothing and $\alpha$ estimates and are summarized in Table 1.

#### Exemplar representation

Participants learned the exemplars’ screen locations and criterion values well. On average, they needed six blocks (*SD* = 3) to learn the locations, which they correctly recalled with 89% accuracy at the end of the experiment. Similarly, participants learned the criterion values within seven blocks (*SD* = 2) and continued to judge the exemplars correctly in the test phase with 67% accuracy. Location accuracy and criterion accuracy were related, $\tau$ = .35, *p* = .002. As location accuracy is only vaguely graded (eight responses per subject), we combined it with the criterion accuracy by computing the mean of the two measures by participant (see *exemplar accuracy* in Table 1).

#### Looking-at-nothing

After the test stimulus removal, a participant looked at blank exemplar locations in *M* = 38% of the trials (*SD* = 32 percentage points) and clicked to reach the response scale after *M* = 3760 ms (*SD* = 2822 ms). We computed looking-at-nothing as the summed gaze duration to the exemplar locations divided by the summed gaze duration to the exemplar and test stimulus locations (*looking-at-nothing duration* in Table 1)^4^. The mean looking-at-nothing is .19 and varies considerably across participants (*SD* = .20), but is comparable when the test stimulus was old (*M* = .17) or new (*M* = .19), $$\tau $$ = .75, $$p < $$ .001. Looking-at-nothing is quite stable within participants, with stimulus-wise split-half correlations between odd and even trials of *M*= .81 (*SD* = .05 across participants). Furthermore, looking-at-nothing is associated with a higher exemplar accuracy after training, $$\tau $$ = .34, $$p < $$ .001 (see Table 1). Thus, especially the participants who learned the exemplars well gazed back at them in the test phase.

#### Association between looking-at-nothing and cognitive modeling

**Fig. 3:**
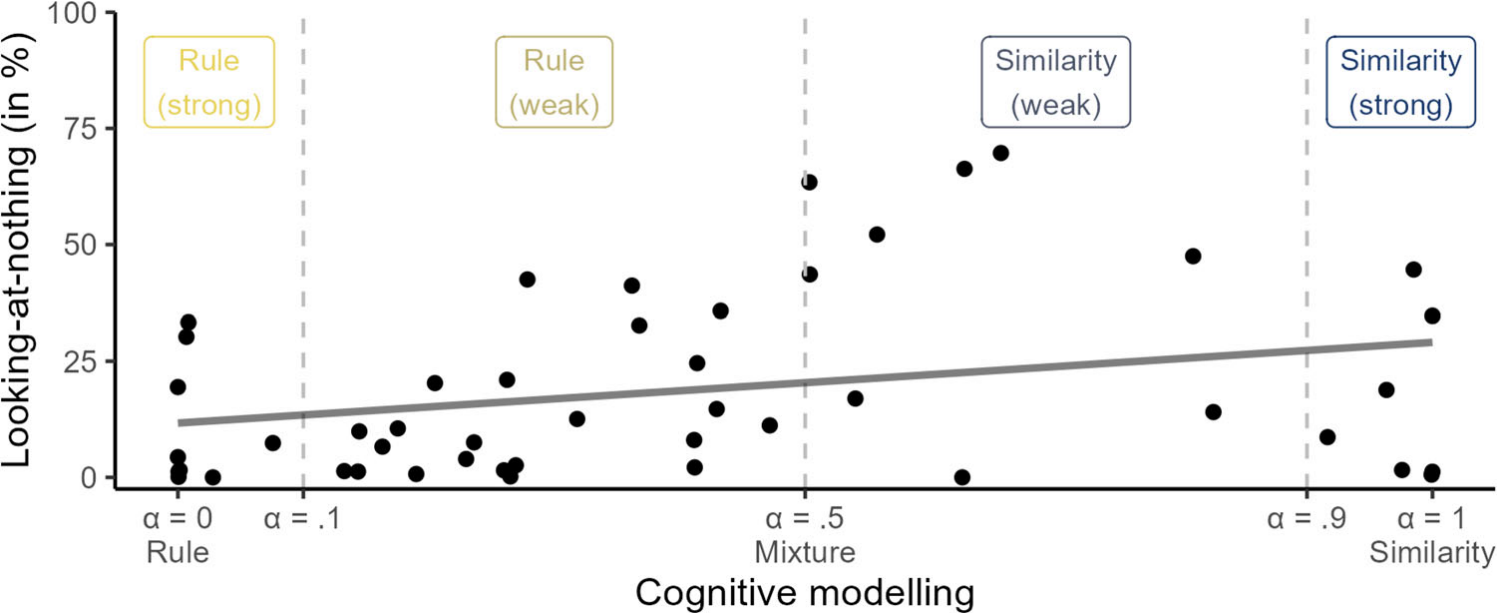
Association between parameter $\alpha$ and looking-at-nothing. Each point shows the mean estimated $\alpha$ value and the mean looking-at-nothing duration of one participant. A larger $\alpha$ is associated with more looking-at-nothing (*dark gray regression line*), in particular for the participants who only weakly rely on one of the two processes (.1 < $\alpha$ < .9). Note that the precise location of these cutoffs is somewhat arbitrary and was defined post hoc to illustrate differences for participants who follow a cognitive process to a weaker or stronger extent

The estimated $$\alpha $$ values vary substantially (*SD* = .32) with a tendency for more rule use, *M* = .40 $$\ne .50$$, *t*(47) = -2.17, 95% CI = [.31, .49], *p* = .04 (see Table 1; for the remaining parameter estimates, see Table 3). Figure 3 shows that participants’ mean $$\alpha $$ values correlate positively with their mean looking-at-nothing: A participant who relied more on similarity over rules according to cognitive modeling (a larger $$\alpha $$) also displayed more looking-at-nothing, $$\tau $$ = 0.25, *p* = .01 (see Table 1). The association was particularly pronounced for the 32 participants who weakly relied on a rule or similarity (.1 < $$\alpha $$ < .9), $$\tau $$ = 0.43, $$p < $$ .001, with a drop in looking-at-nothing among the participants with $$\alpha $$ > .9.

**Table 2:** Looking-at-nothing and mean responses to critical stimuli for rule users and similarity users

		Looking-at-nothing		Mean responses to critical stimuli			
Group	*N*	Duration	Strength	11	12	34	44
Rule users	32	.13	.13	3.13	5.70	19.56	25.30
				(-0.87)	(+0.70)	(-2.44)	(-3.70)
Similarity users	16	.30	.34	4.64	8.15	13.73	21.78
				(+0.64)	(+3.15)	(-8.27)	(-7.22)

*Note.* Rule users are defined by $\alpha$ < .5; similarity users by $\alpha$ > .5. As in Table 1, the looking-at-nothing duration considers participants’ gaze duration to all exemplar locations, the strength only the gaze duration to the location of the most similar exemplar. The critical stimuli discriminate between rule- and similarity-based processes (see Fig. 1). For instance, stimulus 11 with a true criterion value of 4 is underestimated by the rule users by -0.87 units on average, but overestimated by the similarity users by +0.64 units

On the one hand, this finding might suggest a non-linear relationship between $\alpha$ and looking-at-nothing: Participants who strongly rely on similarity might have encoded the exemplars in a particularly robust way and no longer need to look at their locations during retrieval. However, exemplar accuracy did not differ between the seven participants with $\alpha$ > .9 (*M* = 90%) and the nine participants with .5 < $\alpha$ < .9 (*M* = 88%), tie-corrected asymptotic Wilcoxon–Mann–Whitney test *W* = 28, *p* = .75.

On the other hand, the result may have been influenced by a subgroup of four participants who generalized the exemplars’ criterion values to all objects with the same value on one cue, regardless of the other cue value. Our modeling described these participants by an almost maximal $$\alpha $$ (*M* = .99) with the weight $$\beta _n$$ of the ignored cue *n* approaching zero^5^. Such a single-cue strategy no longer requires retrieving complete exemplars, but only the combination of a cue value and the criterion value, relaxing the dependency on exemplar memory. Accordingly, the four participants displayed rather low looking-at-nothing (*M* = 10%) – removing them increased the correlation between $$\alpha $$ and looking-at-nothing to $$\tau $$ = 0.37, $$p < $$ .001.

We further corroborated our analyses by comparing the 32 rule users with $\alpha$ < .5 and the 16 similarity users with $\alpha$ > .5 (see Table 2). The similarity users tended to display more looking-at-nothing (*M* = 30%) than the rule users (*M* = 13%), *W* = 160.5, *p* = .04, in particular when the four similarity users who ignored one cue are excluded (*M* = 37%), *W* = 77.5, *p* = .003. Furthermore, the two groups responded differently to the critical stimuli with extreme cue values that distinguish best between the two processes (stimuli 11, 12, 34, and 44, see Fig. 1). Relative to the rule users, the similarity users made much more moderate judgments that do not extrapolate beyond the range of the exemplars’ criterion values learned during training, leading to larger judgment errors (see Table 2). Across all stimuli, however, the cognitive process used was not associated with the judgment accuracy: The correlation between $\alpha$ and the mean absolute judgment error is $\tau$ = 0.10, *p* = .31, and the mean absolute judgment error does not differ among rules users (1.71) and similarity users (1.89), *W* = 178, *p* = .09.

#### Properties of looking-at-nothing

**Fig. 4:**
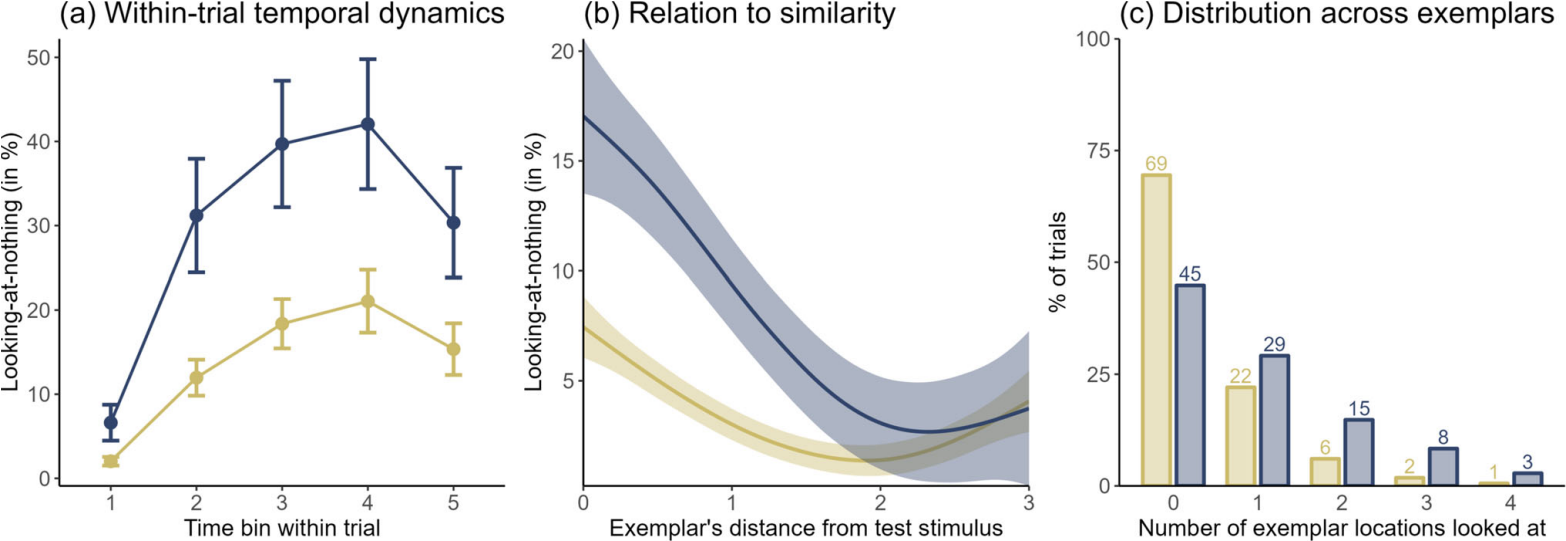
Properties of looking-at-nothing for similarity users ($\alpha$ > .5, shown in *blue*) and rule users ($\alpha$ < .5, shown in *yellow*). (**a**) The looking-at-nothing (mean and standard error) when the trial time after the removal of the test stimulus is evenly split into five bins. (**b**) The smoothed looking-at-nothing (mean and 95% CI) as a function of the exemplar’s weighted city-block distance from the test stimulus (using each participant’s median estimated cue weights $\beta _1$ and $\beta _2$, normalized by participant to sum to 1). (**c**) How many exemplar locations were looked at in how many percent of the trials

Beyond analyzing the quantitative association between $\alpha$ and looking-at-nothing, our multi-method approach of cognitive modeling and eye tracking allows for qualitative insights into people’s judgment formation process. Figure 4 shows the key results.

Figure 4(a) shows how looking-at-nothing unfolded within trials after the test stimulus was removed. To this end, the remaining trial time was split into five bins of equal length (*M* = 756 ms and *SD* = 1119 ms across trials), and participants’ mean looking-at-nothing duration was computed for each bin (further aggregated across participants grouped into similarity users, $\alpha$ > .5, and rule users, $\alpha$ < .5, in Fig. 4). Looking-at-nothing peaked halfway through a trial (bin 4; $M_{\text {similarity}}$ = 42% among the similarity users > $M_{\text {rule}}$ = 21% among the rule users, *W* = 147.5, *p* = .02) and was lowest in bin 1, where participants still fixated the screen center where the test stimulus had just disappeared ($M_{\text {similarity}}$ = 7% $\approx$ $M_{\text {rule}}$ = 2%, *W* = 191, *p* = .16). Participants tended to gaze back at the screen center shortly before they reached the response scale (bin 5; $M_{\text {similarity}}$ = 30% $\approx$ $M_{\text {rule}}$ = 15%, *W* = 168.5, *p* = .06); however, the difference to bin 4 was not significant, *W* = 953, *p* = .15 across all participants.

Furthermore, looking-at-nothing increased with the exemplar’s similarity to the test stimulus – in particular for the participants classified as similarity users (see Fig. 4b). Looking-at-nothing peaked when an exemplar matched the test stimuli (a difference of 0 in Fig. 4; $$M_{\text {similarity}}$$ = 20% > $$M_{\text {rule}}$$ = 8%, *W* = 160, *p* = .04) and decreased the more an exemplar differed from the test stimulus in terms of the city-block distance, $$\tau $$ = -.22, $$p < $$ .001 for the similarity users and $$\tau $$ = -.10, $$p < $$ .001 for the rule users. Similarity users showed more looking-at-nothing than rule users for exemplars whose city-block distance from the test stimulus was lower than 1 ($$M_{\text {similarity}}$$ = 11% > $$M_{\text {rule}}$$ = 4%, *W* = 144.5, *p* = .02) but not for exemplars with a city-block distance larger than 1 ($$M_{\text {similarity}}$$ = 4% $$\approx $$ $$M_{\text {rule}}$$ = 2%, *W* = 194.5, *p* = .18). Thus, the similarity users particularly focused on exemplars similar to the test stimulus. This finding is corroborated by the so-called *looking-at-nothing strength* (i.e., the gaze duration to the most similar exemplar divided by the summed gaze duration to all exemplar locations): The looking-at-nothing strength is larger for the similarity users (*M* = 34%) than for the rule users (*M* = 13%), *W* = 137.5, *p* = .01 (see Table 2), and positively correlates with *alpha*, $$\tau $$ = .33, *p* = .001 (see Table 1).

Finally, Fig. 4(c) shows that looking-at-nothing was present in *M* = 55% of trials among the similarity users, but only in *M* = 31% of trials among the rule users, $$\chi $$^2^(1) = 338.74, $$p < $$ .001. In these looking-at-nothing trials, participants usually only looked at one exemplar location, $$M_{\text {rule}}$$ = 72% > $$M_{\text {similarity}}$$ = 53%, $$\chi $$^2^(1) = 92.72, $$p < $$ .001. The location being looked at often corresponded to the exemplar most similar to the test stimulus, especially for the similarity users (*M* = 76%) and, to a lesser extent, for the rule users (*M* = 46%), $$\chi $$^2^(1) = 128.94, $$p < $$ .001. Even when participants looked at multiple exemplar locations within trials, most looking-at-nothing time was directed at a single location (*M* = 66%, *SD* = 15 percentage points) which belonged to the most similar exemplar in *M* = 48% of the trials ($$M_{\text {similarity}}$$ = 53% > $$M_{\text {rule}}$$ = 41%, $$\chi $$^2^(1) = 10.21, *p* = .001). Accordingly, the number of exemplar locations looked at was only weakly related to the reaction time, $$\tau $$ = .07, $$p < $$ .001, with every additional location increasing the reaction time by about 23 ms, *t* = 5.7, $$p < $$ .001. This suggests that people often judge an object’s criterion based on a single, similar exemplar, in line with Albrecht et al. (2020).

The properties of looking-at-nothing differ little between old and new test stimuli, in particular for the rule users who displayed looking-at-nothing in 31% of trials with a new test stimulus and 30% of trials with an old test stimulus, $\chi$^2^(1) = 0.17, *p* = .68. The similarity users, in turn, tended to look at the blank exemplar locations in more trials when the test stimulus was new rather than old, 57% > 51%, $\chi$^2^(1) = 5.31, *p* = .02. The within-trial temporal dynamics followed virtually an identical pattern for old and new test stimuli, again in particular for the rule users. The similarity users tended to exhibit slightly higher looking-at-nothing durations within trials when the test stimulus was new rather than old, without, however, reaching significance, *W* = 3609, *p* = .16. In turn, the looking-at-nothing strength was particularly high for the old test stimuli, indicating that participants looked back briefly at the corresponding exemplar with old test stimuli, but considered multiple exemplar locations more evenly with new test stimuli.

## General discussion

This work used a multi-methodological approach to investigate the cognitive processes involved when people infer an object’s unknown criterion value from its cues. Analyzing process and behavioral data from a multiple-cue judgment task, we found a clear correspondence between the results obtained from cognitive modeling and eye tracking: The more cognitive modeling indicated reliance on the similarity to exemplars, the more participants looked at the blank locations where the exemplars were previously encoded (looking-at-nothing, Scholz et al., 2015). Further analyses showed that looking-at-nothing peaked halfway through a trial, increased for exemplars more similar to the test stimulus being judged, and focused mostly on one exemplar per trial.

We observed overall relatively low levels of looking-at-nothing. This may have methodological reasons: First, looking-at-nothing occurs more often when the stimuli are content-rich and concrete rather than abstract (e.g., job application profiles instead of geometrical figures) and when the test stimuli are presented in an auditory way (Rosner et al., 2022). The sequential presentation of auditory test stimuli’s cues takes much encoding time and increases the fixation proportions for all exemplar locations. Second, other studies maximized exemplar recall by instructing a corresponding strategy (in contrast to the present studies), thus also boosting looking-at-nothing (Scholz et al., 2015). Finally, looking-at-nothing increases when the test stimuli are only shown briefly, which was also the case in our studies, leading to comparable occurrence rates as in Rosner et al. (2022, Exp. 1b), who removed visually presented, conceptual test stimuli after 1.5 s.

Comparing the two studies, we found substantially more looking-at-nothing and more similarity use in the multiplicative environment reported in the main text than in the additive environment reported in Appendix B. This is consistent with a cognitive perspective: Additive environments can be perfectly learned by rule-based processes (which assume an additive relation between cues and criterion, see App. A) but not by similarity-based processes. Accordingly, the human mind has a strong inclination to use a rule in an additive environment (Hoffmann, von Helversen, & Rieskamp, 2016; Juslin et al., 2008; Karlsson et al., 2007) and may therefore display little looking-at-nothing (Scholz et al., 2015).

While relying on the similarity to exemplars seems to be clearly associated with looking back at the blank exemplar locations, the present work reported only correlational evidence. The causal direction thus remains unclear: Previous research has shown that inducing either a rule or a similarity process affects looking-at-nothing accordingly (Scholz et al., 2015); however, more looking-at-nothing might also lead to more exemplar retrieval and similarity use. Future research could test this hypothesis by experimentally manipulating people’s eye movements to the blank exemplar locations.

**Fig. 5:**
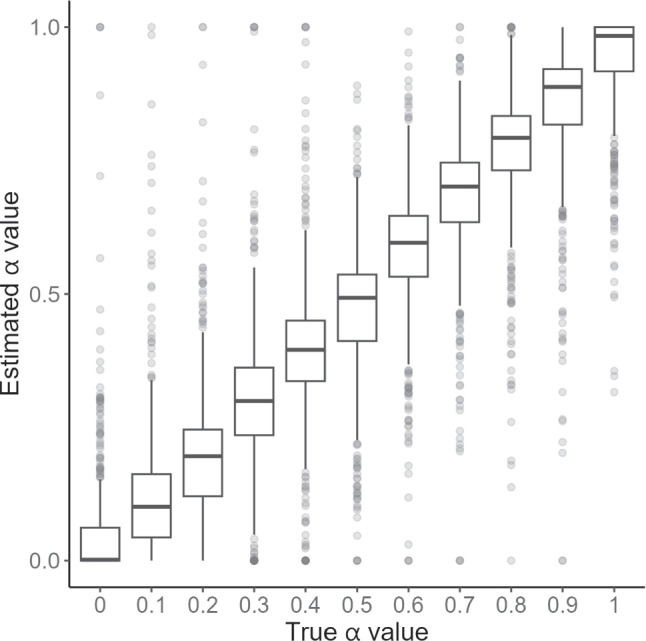
Parameter recovery. For each true $\alpha$ level, the $\alpha$ values estimated from simulated responses in our experimental task are shown as box plots over the 500 simulation iterations

Furthermore, while our application of cognitive modeling filled a gap in previous literature on looking-at-nothing and exemplar retrieval, the frequentist modeling framework we used can bias $\alpha$ towards the rule process in the presence of much noise (Bröder et al., 2017; Izydorczyk & Bröder, 2022). We accepted this as we focused on the association between $\alpha$ and looking-at-nothing, which remains unaffected by linear transformations in the $\alpha$ estimates. Also, parameter $\alpha$ was well recovered in our task even in the presence of noise (see Fig. 5 in the Appendix), further corroborating the validity of our approach.

Zooming out, our results suggest that cognitive modeling and process tracing provide mutually supportive insights into the processes underlying higher-order cognition. Process tracing may be particularly beneficial when different cognitive processes lead to similar response predictions and thus cannot be distinguished at the behavioral level (e.g., when test stimuli do not extrapolate beyond the range of the exemplars’ criterion values). Note that other process tracing methods than eye tracking can provide important insights into the judgment formation processes too (e.g., verbal reports, Steiner, Seitz, & Frey, 2021). One avenue for future research could be to broaden up the multi-method approach of this paper by comparing multiple process tracing methods with cognitive modeling.

## Conclusion

We found that the process people rely on according to cognitive modeling to make inferences is reflected in their eye movements. Fixating the locations of previously encoded exemplars is associated with a cognitive process that relies on the similarity to exemplars. Additionally, the synergy of cognitive modeling and process tracing brings qualitative insights into the exemplar retrieval. Ultimately, the multi-method approach of the present work sheds light on the processes underlying human cognition in a way that either approach in isolation would be hardly able to do.

## Figures and Tables

**Fig. 6:**
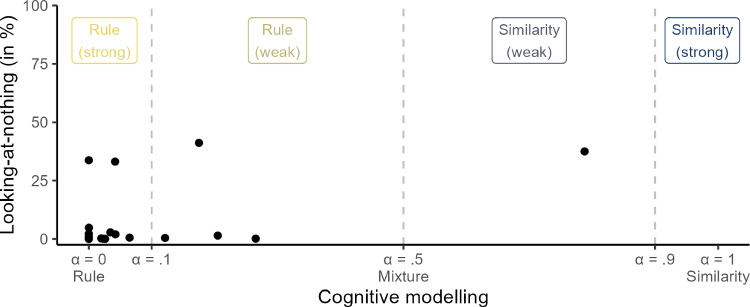
No association between $\alpha$ and looking-at-nothing in the additive experiment. Each *point* shows the mean estimated $\alpha$ value and the mean looking-at-nothing of one participant

**Fig. 7:**
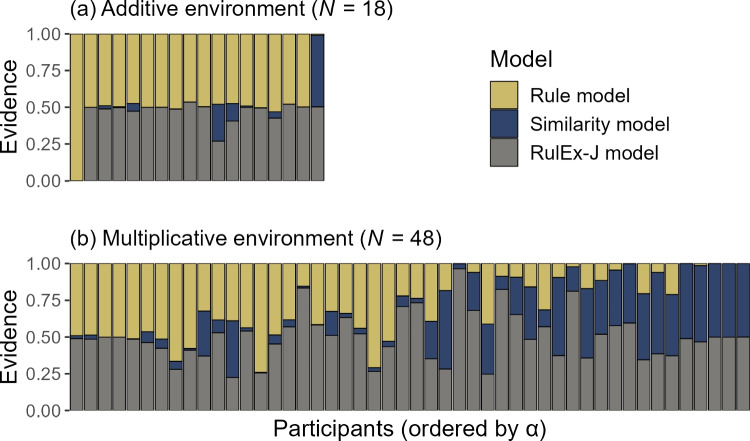
Evidence strengths (Akaike weights) of RulEx-J, a rule model, and a similarity model in the additive (**a**) and multiplicative studies (**b**). Each participant is one bar, and the evidence for the different models is stacked on top of each other, summing to 1 for each participant. The *x*-axis orders participants by their $\alpha$ value from smallest (*left*) to largest (*right*)

## Data Availability

The anonymized data, the complete analysis code, and the materials are available at https://osf.io/k82yt/. None of the experiments was preregistered.

## Appendix A. Cognitive models

We performed cognitive modeling within the RulEx-J framework of Bröder et al. (2017), which predicts people’s multiple-cue judgment to be a weighted average of the predictions resulting from a similarity- and a rule-based process (see Eq. 1). We now outline the formal similarity- and rule-based processes we used, which are based on Juslin et al. (2003).

### Similarity-based process

The similarity-based process predicts an object’s criterion value based on its similarity to previously experienced objects called exemplars. Similar exemplars affect the object’s predicted criterion value more than dissimilar exemplars. Specifically, the similarity-based process predicts object *i*’s criterion value $\hat{c}_i^{\text {Similarity}}$ to be the mean of the criterion values across exemplars, each weighted by its normalized similarity to *i*. Formally,

A1 $$\begin{aligned} \hat{c}_i^{\text {Similarity}} = \frac{\sum _{j} s_{ij} \cdot c_j}{\sum _{j} s_{ij}}, \end{aligned}$$

where $s_{ij}$ is the similarity between object *i* and exemplar *j* with criterion value $c_j$.

The similarity $s_{ij}$, in turn, is computed from the summed cue value differences between object *i* and exemplar *j*. A larger difference results in a smaller similarity and vice versa. We modeled the relation between the similarity $s_{ij}$ and the summed difference $d_{ij}$ with Shepard’s (1987) negative exponential function $s_{ij} = \exp \left( -d_{ij}\right)$ and computed $d_{ij}$ with the weighted city-block distance $d_{ij} = \sum \limits _{n = 1}^N \beta _n \cdot |x_{in} - x_{jn}|$, leading to

A2 $$\begin{aligned} s_{ij} = \exp \left( -\sum \limits _{n = 1}^N \beta _n \cdot |x_{in} - x_{jn}|\right) , \end{aligned}$$

where $x_{in}$ is object *i*’s value *x* on cue *n* and $\beta _n$ is a free model parameter denoting the weight given to the differences between object *i* and exemplar *j* with respect to cue *n* (with $\beta _n \ge 0$). Equation A2 thus computes a weighted sum of the cue value differences and transforms it into an inversely related measure of similarity. Other distance-based formalizations for $s_{ij}$ exist (see Nosofsky, 1986; Seitz, 2024) – the city-block distance describes the similarity perceived by people between stimuli with highly separable cues such as ours particularly well (Shepard, 1964).

Our formalization follows Albrecht et al. (2020). An alternative, equivalent formalization constrains the weights to sum to 1 ($\sum _n \beta _n = 1$) and adds a scaling parameter *c* to the negative exponential function, $s_{ij} = \exp \left( -c \cdot d_{ij}\right)$. Parameter *c* defines how steeply similarity declines with increasing distance and can be interpreted in terms of the number of exemplars the similarity-based process relies on (see Collsiöö, Sundh, & Juslin, 2023). This alternative version can be recreated from our version by setting the scaling parameter *c* to the sum of the weights $\sum _n \beta _n$ and by normalizing the weights to sum to 1 (Seitz, 2023). Our formalization, however, maximizes the comparability between the weights in the similarity-based process and the weights in the rule-based process, introduced below.

### Rule-based process

In contrast, the rule-based process uses a main effects linear regression model to predict an object’s criterion value from its cues. Specifically, the rule-based process predicts object *i*’s criterion value $\hat{c}_i^{\text {Rule}}$ as a weighted sum of *i*’s cue values

A3 $$\begin{aligned} \hat{c}_i^{\text {Rule}} = \beta _0 + \sum \limits _{n=1}^N \beta _n \cdot x_{in}, \end{aligned}$$

where $\beta _0$ represents an intercept (i.e., the predicted criterion value when all cue values equal zero) and $\beta _n$ represents the weight for cue *n*. We constrained the weight $\beta _n$ in the rule-based process to equal $\beta _n$ in the similarity-based process, as this model led to a higher out-of-sample log-likelihood.

### Combining similarity- and rule-based processes: Parameter recovery of $\alpha$

We combined the similarity- and rule-based processes using the RulEx-J framework of Bröder et al. (2017), $\hat{c}_i = \alpha \cdot \hat{c}_i^{\text {Similarity}} + (1 - \alpha ) \cdot \hat{c}_i^{\text {Rule}}$ (Eq. 1), and assumed that people’s judgments are sampled from a normal distribution, $R_i \sim \mathcal {N}\left( \hat{c}_i, \sigma \right)$, where the mean equals the combined prediction $\hat{c}_i$ and the standard deviation $\sigma \ge 0$ is a free parameter.

Ultimately, we aimed to associate the estimated $\alpha$ values with participants’ eye movement data. To test whether $\alpha$ was recoverable in our experimental task, we simulated multiple-cue judgments based on various parameter value combinations and checked to what extent the estimated $\alpha$ values when fitting the models correspond to those used to simulate the data. Specifically, in each out of 500 iterations, we randomly sampled a parameter value combination from uniform distributions (excluding $\alpha$ and constraining $-10 \le \beta _0 \le 10$, $0 \le \beta _n \le 10$ for $n \in \{1, 2\}$, and $0 \le \sigma \le 10$). The sampled parameter value combination was used to make predictions for the similarity- and rule-based processes as per Eqs. A1 and A3. Then, for each $\alpha$ level from 0 to 1 in steps of .1, the combined prediction $\hat{c}_i$ was computed and used to simulate noisy responses $R_i \sim \mathcal {N}\left( \hat{c}_i, \sigma \right)$ for all 16 stimuli *i* repeated eight times as in the actual experiments. The model was then refitted to the simulated responses using maximum likelihood.

Parameter $\alpha$ was recovered well in our task (see Fig. 5). The mean correlation between the true and estimated $\alpha$ across iterations was $r = 0.92$. The mean absolute error was low (MAE = .09), and the mean signed difference was close to zero (MSD = .01), indicating no estimation bias. An $\alpha$ of .5 (denoting equal reliance on the similarity- and rule-based processes) was well recovered, *M* = .47 across iterations, and a stronger reliance on one process was almost perfectly recovered qualitatively: The true and estimated values of $\alpha$ diverged from .5 in the same direction in 93% of cases. This means that we can classify participants into rule and similarity users with confidence based on their $\alpha$ value.

### Summary

Our cognitive modeling combines a similarity- and a rule-based process (see Juslin et al., 2003) using the RulEx-J framework of Bröder et al. (2017). In our task with $N = 2$ cues, this yields five free model parameters: the intercept $\beta _0$, the weights $\beta _1$ and $\beta _2$, $\alpha$ denoting the reliance on similarity over rules, and the standard deviation $\sigma$ of the normal distribution relating predictions to responses. Given that in our task the feature values ranged from 1 to 4 and the criterion values from 0 to 30, we constrained the parameters as follows: $-10 \le \beta _0 \le 10$, $0 \le \beta _n \le 10$ for $n \in \{1, 2\}$, $0 \le \alpha \le 1$, and $0 \le \sigma \le 10$.

## Appendix B. Study with an additive environment

This section shows the result of the study using an additive function to link the cues to the criterion. Compared to the multiplicative environment, the additive environment yielded only little looking-at-nothing and little reliance on similarity, which is in line with previous research (e.g., Hoffmann et al., 2016; Juslin et al., 2008; Karlsson et al., 2007).

### Method

The method was the same as in the multiplicative environment, except that (i) the additive function $c = 5 \cdot x_1 + x_2$ was used; (ii) the stimuli 13, 24, 32, and 41 were selected as exemplars; and (iii) the final sample included *N* = 18 participants (12 women; age: *M* = 21 years, *SD* = 7 years; one additional participant did not complete the experiment due to eye-tracking problems). The experiment was discontinued at *N* = 18 participants, because only little looking-at-nothing and similarity use were observed. More variability in both measures would have been needed for reliable estimates of the association between them.

### Results

#### Exemplar representation

As in the multiplicative environment, participants learned the exemplars well, the locations within *M* = 4 blocks (*SD* = 1) and the criterion values within *M* = 6 blocks (*SD* = 2). The accuracy remained high after training, with *M* = 94% (*Mdn* = 100%, *SD* = 12 percentage points) for the locations and *M* = 80% (*Mdn* = 95%, *SD* = 28 percentage points) for the criterion values. As accuracy was generally very high, no association between location and criterion accuracy was found, $\tau$ = -.05, *z* = -0.22, *p* = .83.

#### Looking-at-nothing

The test stimuli were removed after *M* = 371 ms (*SD* = 222 ms), and participants reached the response scale after another *M* = 3184 ms (*SD* = 2436 ms). Only *M* = 20% of the trials (*SD* = 31 percentage points) contained looking-at-nothing^6^, and a participant’s average looking-at-nothing duration across trials was only *M* = 9% (*Mdn* = 1%, *SD* = 15 percentage points across participants). Looking-at-nothing was comparable for old and new test stimuli ($$M_{\text {old}}$$ = 10% $$\approx $$ $$M_{\text {new}}$$ = 9%, $$\tau $$ = .65, *z* = 3.58, $$p < $$ .001) and was stable within participants, with stimulus-wise split-half correlations of *M*= .81 (*SD* = .12). Thus, most participants consistently showed no looking-at-nothing.

#### Association between looking-at-nothing and cognitive modeling

The estimated values for $$\alpha $$ were low (*M* = .10, *SD* = .19), indicating strong rule use, *V* = 2, $$p < $$ .001. Six participants had an $$\alpha $$ = 0 (pure rule users), and only one participant had an $$\alpha $$ > .5. This similarity user also showed substantial looking-at-nothing (*M* = 38%). However, across participants, there was no credible association between their $$\alpha $$ estimates and mean looking-at-nothing, $$\tau $$ = 0.10, *p* = .60. One reason for this is the insufficient variability both in $$\alpha $$ and looking-at-nothing, illustrated in Fig. 6.

## Appendix C. Model comparison

To further test the validity of the $\alpha$ parameter combining a rule and a similarity-based process, we compared the RulEx-J model to a pure similarity model and a pure rule model. Both models were identical to RulEx-J, except that we fixed $\alpha$ = 0 for the rule model and $\alpha$ = 1 for the similarity model (which thus additionally did not have the intercept parameter $\beta _0$). As for RulEx-J, all parameters were estimated by maximum likelihood using the 16-fold cross-validation, in which each test stimulus was once used for out-of-sample prediction. The parameter estimates were averaged by participant across the 16 folds with the mean, and Table 3 shows the distribution of the resulting values across participants.

**Table 3:** Mean model parameter estimates (standard deviation in brackets) across participants

	Additive environment (*N* = 18)					Multiplicative environment (*N* = 48)				
Model	$\alpha$	$\beta _0$	$\beta _1$	$\beta _2$	$\sigma$	$\alpha$	$\beta _0$	$\beta _1$	$\beta _2$	$\sigma$
RulEx-J	0.10	-0.99	4.72	1.81	1.56	0.40	-5.58	3.72	3.72	2.79
	(0.19)	(3.57)	(1.35)	(1.10)	(1.37)	(0.32)	(5.68)	(1.84)	(2.02)	(1.67)
Rule	-	-0.47	4.71	1.57	1.60	-	-3.93	3.73	2.81	3.40
		(3.47)	(1.18)	(1.02)	(1.39)		(5.08)	(1.57)	(1.22)	(1.59)
Similarity	-	-	8.64	0.06	2.86	-	-	5.71	6.03	3.94
			(2.65)	(0.20)	(1.69)			(3.01)	(2.76)	(1.87)

*Note.* The standard deviations are computed across participants, using each participant’s mean parameter estimates across folds. The similarity model does not contain parameter $\beta _0$ and implicitly assumes $\alpha$ = 1, while the rule model implicitly assumes $\alpha$ = 0

**Table 4:** Descriptive out-of-sample model fit measures aggregated across participants

	Additive environment ($N = 18$)					Multiplicative environment ($N = 48$)				
Model	*w*_AIC_	*M* $_{\ell }$	*SD* $_{\ell }$	*MAE*	*RMSE*	*w*_AIC_	*M* $_{\ell }$	*SD* $_{\ell }$	*MAE*	*RMSE*
RulEx-J	.45	-3.67	28.77	1.19	1.71	.50	-15.83	11.65	2.47	3.19
Rule	.49	-3.41	30.61	1.20	1.72	.29	-19.75	4.74	3.19	3.91
Similarity	.06	-16.71	4.55	2.22	2.90	.21	-18.12	11.74	3.19	4.22

*Note.* Fit measures are computed at the aggregate level: *w*_AIC_ = mean evidence strength based on Akaike weights, $\ell$ = participant-wise median out-of-sample log-likelihood across folds (summarized across participants with the mean *M*$_{\ell }$ and the standard deviation *SD*$_{\ell }$), *MAE* = mean absolute error, *RMSE* = root mean square error

To study the out-of-sample performance, we computed the log-likelihoods from the out-of-sample predictions of the RulEx-J model, the similarity model, and the rule model. For each participant and model, we transformed the median out-of-sample log-likelihood across folds into a model evidence strength (i.e., Akaike weight, Wagenmakers & Farrell, 2004). Table 4 reports the mean evidence strengths across participants and additional fit indices; Figure 7 displays the individual evidence strengths. For almost every participant, there is substantial evidence for RulEx-J, but also for one of the alternative models, namely the rule model for the participants with $\alpha$ < .5 on the left, and the similarity model for the participants with $\alpha$ > .5 on the right (this concerns only one participant in the additive environment, Fig. 7a, but multiple participants in the multiplicative environment, Fig. 7b).

## Footnotes

1 In related categorization research, an alternative approach relies on the similarity to a category’s central tendency called prototype (for a comparison of exemplar and prototype models, see Nosofsky & Zaki, 2002).

2 Note, that there exists no direct power analysis for Kendall’s $\uptau$; we therefore ran the analysis for the related rank correlation coefficient (Spearman’s $\uprho$). Smaller observed correlations indicate a lack of power.

3 Each fold used 15 of the 16 stimuli for parameter estimation (120 trials per participant) and the remaining stimulus for out-of-sample prediction (eight trials).

4 The results remain qualitatively unchanged if, instead, normalization is carried out over the total trial duration from removing the test stimulus to reaching the response scale. The two measures of looking-at-nothing are strongly correlated, $$\tau $$ = .91, $$p < $$ .001.

5 The $\alpha$ estimates are not perfectly 1 because the four participants deviated from this single-cue strategy in *M* = 1% of the trials.
